# Combined Dynamic Time Warping with Multiple Sensors for 3D Gesture Recognition

**DOI:** 10.3390/s17081893

**Published:** 2017-08-17

**Authors:** Hyo-Rim Choi, TaeYong Kim

**Affiliations:** Department of Advanced Imaging Science, Chung-Ang University, Heukseok-dong, Dongjak-gu, Seoul 156-756, Korea; funappear@nate.com

**Keywords:** multiple sensors, dynamic time warping, gesture recognition, 3D gesture

## Abstract

Cyber-physical systems, which closely integrate physical systems and humans, can be applied to a wider range of applications through user movement analysis. In three-dimensional (3D) gesture recognition, multiple sensors are required to recognize various natural gestures. Several studies have been undertaken in the field of gesture recognition; however, gesture recognition was conducted based on data captured from various independent sensors, which rendered the capture and combination of real-time data complicated. In this study, a 3D gesture recognition method using combined information obtained from multiple sensors is proposed. The proposed method can robustly perform gesture recognition regardless of a user’s location and movement directions by providing viewpoint-weighted values and/or motion-weighted values. In the proposed method, the viewpoint-weighted dynamic time warping with multiple sensors has enhanced performance by preventing joint measurement errors and noise due to sensor measurement tolerance, which has resulted in the enhancement of recognition performance by comparing multiple joint sequences effectively.

## 1. Introduction

Technological advances in computing, communications, and control have enabled the convergence of physical components and cyberspace. Cyber-physical systems (CPSs), including software, electronic hardware, sensors, actuators, and embedding systems, are the interface between humans and machines and other systems. For further advancement of CPSs, it is necessary for other technologies to be involved, such as advanced interface technologies.

The advancement of sensor technology and machine learning has facilitated a number of studies on methods to replace the traditional interfaces, which use keyboards, the mouse, and control panels, with those that recognize users’ natural motions [1,2]. Specifically, since the release of Kinect, which is a low-priced depth camera, increasing interest has been drawn to the development of gesture recognition methods that can capture gestures more accurately [3]. In addition, studies have been carried out on the performance of depth cameras and articulated objects with depth data wherein the experiment was conducted indoors [4,5].

On comparing Kinect with other cameras used in capturing depth data, such as point grey bumblebee XB3 and Camcube (regardless of the Camcube´s accuracy in depth data capture), it has been determined that in terms of flexibility and price, Kinect is the optimum option for capturing depth data [6].

To enhance the reliability of the data acquired from the depth camera and to obtain various complicated gestures, a number of studies have been carried out on three-dimensional (3D) expression and data correction methods. The observations of these studies have resulted in the accurate recognition of user gestures and more complicated gestures in diverse environments [7,8]. In recent years, the domain of gesture recognition-related studies has been expanded to include virtual reality (which is currently attracting significant interest) and to smart homes based on context awareness. In a number of dynamic environments, it is not convenient to capture user’s movement using a single sensor owing to performance limitation and overlapping. Hence, multiple sensors can provide optimum solutions to such challenges.

In correcting a user’s movements captured using Kinect sensors, there exists a domain where it is not feasible to correct data owing to the location and direction of the subject. Furthermore, measurement errors occur owing to the observation direction of the Kinect camera sensor. Measured values of depth from the overlooking angle of the sensor contain the widest margin of error [4]. This causes a margin of error in the user’s joint information that is extracted from the depth data [9]. Therefore, multiple sensors or additional equipment are required. In this study, gesture recognition algorithms with multiple sensors are proposed in consideration of the viewpoint-weighted values and motion-weighted values to develop a 3D gesture recognition algorithm that is applicable to various environments. In the proposed method, the gesture recognition accuracy is enhanced by providing a higher-weighted value to the data with a lower margin of error while considering movement directions and camera locations. Furthermore, it has been determined necessary to differentiate the significance of each joint in order to recognize the gestures that involve the movement of multiple joints [10]. The proposed method enables this if higher weight values are assigned to joints that require more dynamic movements to differentiate the criticality of the joints.

In the present study, this challenge is circumvented by capturing the gesture regardless of the location through camera sensors, by considering the viewing points of the gesture while calculating the motion weight, and by applying dynamic time warping algorithms to calculate the appropriate similarities.

## 2. Gesture Recognition and Dynamic Time Warping

### 2.1. Gesture Recognition

For recognition of user gestures, studies have been carried out on various pattern recognition algorithms including dynamic time warping (DTW) [11], the hidden Markov model (HMM) [12], and conditional random fields (CRF) [13]. DTW is an algorithm that uses the optimized warping route with dynamic planning method to flexibly compare two sequences. This algorithm is being applied to various fields ranging from voice recognition to gesture recognition and signature.

The HMM is a probabilistic model that uses the transition probability of sequence data. As it is adequate for the modeling of sequence data, it is widely used in the gesture recognition field. CRF is similar to the HMM apart from its use of conditional probability that relaxes the independence assumption and prevents the bias problem [13]. DTW is a matching algorithm that is more explicit than the HMM or CRF; however, it involves high computation costs that increase exponentially as a function of the length of sequences [14,15,16,17]. A majority of extant studies on DTW-based gesture recognition considered a single sequence; therefore, further enhancement is required to effectively process multiple sequences obtained from multiple sensors. However, DTW provides linear flexibility of sequential data obtained from multiple sensors, thereby resulting in enhanced accuracy of gesture recognition.

### 2.2. Dynamic Time Warping

DTW is a matching algorithm that allows for the linear flexibility of a sequence. To compare two sequences (*X* = {*x*_1_, *x*_2_, *…*, *x_N_*} and *Y* = {*y*_1_, *y*_2_, *…*, *y_M_*}), the study set up the matching path *W*. The matching path *W* can be defined as:

(1) $$W=\{w_{1},w_{2},\ldots ,w_{K}\}$$

Equation (2), which is designed to minimize the sum of the total distance of the matching path, calculates the distance between the two sequences:

(2) $$DTW(A,B)=\min\sum_{k=1}^{K}d(w_{k})$$

Herein, the function *d* can be defined as:

(3) $$d(w)=d(n,m)=\|x_{n}-y_{m}\|$$

Considering that a large quantity of data is required to compute Equation (2), a dynamic programming method can evaluate it straightforwardly with few constraints. The distance between the two sequences is calculated using Equation (4) [11]:

(4) D(n,m)=d(n,m)+min[D(n−1,m−1),D(n−1,m),D(n,m−1)]

Herein, Matrix D is a cost matrix that accumulates the distance between the two sequences.

## 3. Dynamic Time Warping with Multiple Sensors

**Figure 1:**
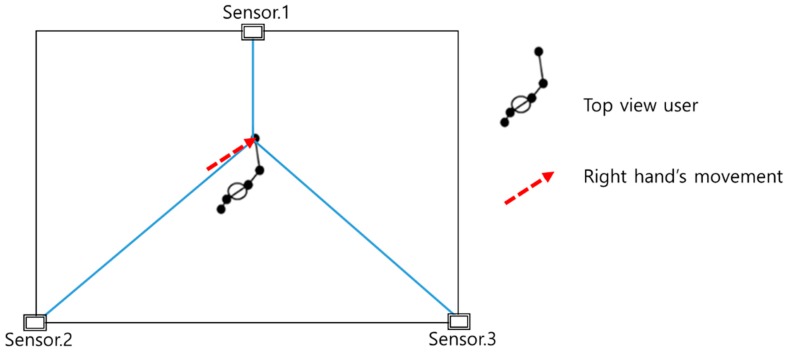
Angles between multiple sensors and user movement trajectory.

This section describes the use of multiple sensors to recognize 3D gestures, which is applicable to various environments, and the introduction of dynamic time warping with multiple sensors (DTWMS) to utilize sequences obtained from the multiple sensors in this study. In this experiment, three to four Kinect sensors, which can cover the entire area of the users’ activities, were installed to observe the movements of users. Figure 1 illustrates the angle between the installed sensors and the users’ movement trajectory.

Figure 1 illustrates that the use of a number of sensors provides the advantage of capturing numerous points in the blind spot. However, owing to the fact that Kinect uses the infrared (IR) point cloud, as the number of sensors increase beyond three, interference occurs between the IR patterns, resulting in malfunction. Therefore, owing to the nature of the proposed method, wherein blind spot resolution and *z*-directional motion enhancement are the main goals, three sensors were observed to yield appropriate results. Therefore, to circumvent this problem in the present experiment, the number of Kinect sensors was reduced to three, and the experiment was conducted in an indoor environment. The cameras were set in an area of 4 m^2^ with 120° angular variation between each camera.

Kinect sensors are capable of collecting users’ movement more effectively than red, green, and blue light (RGB) sensors; however, there is a possibility of the existence of a domain where it is not feasible to collect data, depending on the location and direction of the subject. Furthermore, measurement errors are likely to occur depending on the observation direction of the Kinect sensors; for example, measurement values on the *z*-axis from the overlooking angle of a sensor exhibit the widest margins of error [4]. Owing to this problem, the margins of error can exist in the users’ joint information, which is extracted from the depth data [9]. The proposed method is designed to enhance the gesture recognition accuracy by assigning higher-weighted values to data with a lower margin of error in consideration of joint movement directions and camera locations.

In addition, when a gesture involves whole body movement, the concerned joints might not exhibit an equal level of criticality; therefore, it is necessary to differentiate the significance of each joint to effectively recognize the gestures that are required to perform the movement of multiple joints [10]. The dynamic movement-based comparison can be feasible if higher weight values are assigned to joints that require more dynamic movements to differentiate the criticality of joints.

### 3.1. Multiple Sequence Preprocessing

Considering that the location and overlooking angle of the installed sensors vary, it is necessary to normalize the joint-related data obtained from all the sensors. The normalization of the roll of the obtained data is not required because the cameras are installed on flat spots, while normalization is necessary for pan and tilt.

**Figure 2:**
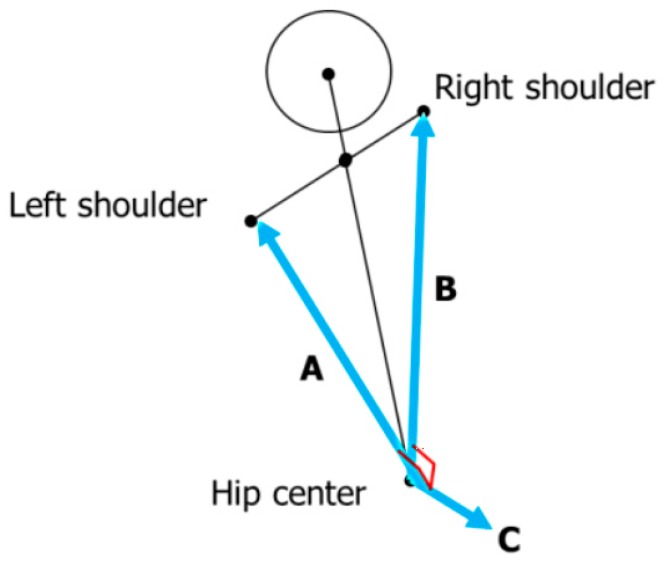
Two vectors used for normalization (A, B) and outer value (C).

Considering the outer value of a joint and two vectors, which connect both the shoulders of a user (A and B in Figure 2), the overlooking direction of a camera is calculated. By using Vector C, the joint information is normalized to adjust the overlooking direction to the front side (pan and tilt at 0).

After normalization (front rotation and adjustment), certain issues, such as left and right decision, arise due to the functionality of the software development kit of a few of the sensors as they were designed for indoor game activities. Therefore, to overcome this, all the sensors joints are considered. Then, the sequence with adequately-tracked joints is considered as the reference sequence, while the remaining sequences from the other sensors are to be decided with respect to the reference joint sequence.

**Figure 3:**
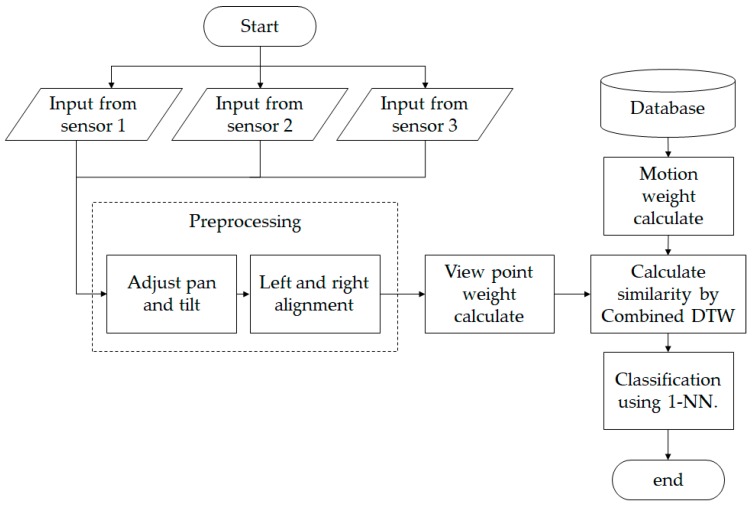
Flow chart of the proposed method. DTW: dynamic time warping. 1-NN: 1-nearest neighbor.

The Figure 3 illustrates that the present experiments were conducted offline. As the purpose of the present experiment was to enhance accuracy, the online environment was not considered as the sequences were manually captured given that an online environment would be beyond the scope of this research. However, using certain methods such as spotting and window sliding before input acquisition, it is feasible to apply this method in real-time gesture recognition.

### 3.2. Viewpoint Weight and Motion Weight

Kinect is a structured-light depth camera that emits a certain pattern of infrared rays, which is then captured by the infrared camera for comparison with the reference pattern; the margins of error thus determined is used to calculate the depth using the triangulation method.

**Figure 4:**
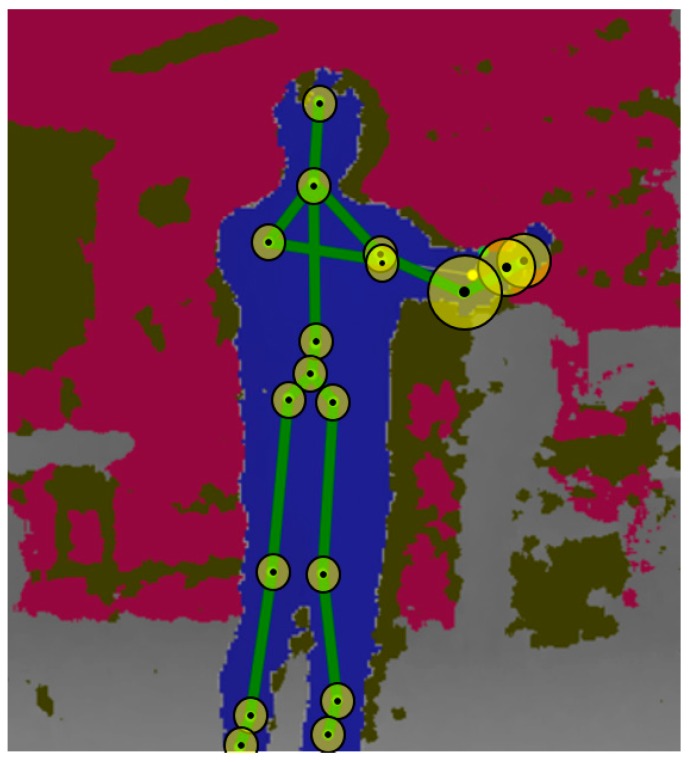
Visual example of different weights on each joint.

As indicated in Figure 4, various visual weights were considered as depicted by the circles on each joint. In this figure, the rear right camera was placed under consideration where the captured weight joints were considered, and by considering the joint state, which is prepared by Kinect SDK, untracked joints were omitted. However, there are still a few errors, as illustrated in Figure 4. Nonetheless, with the advantage of tracking numerous joints, these errors exert negligible effect on the results of the present study.

In this process, margins of error compared to the actual location are likely to occur, and if the distance from the sensor is larger, the margins of error—including the measurement noise—increase. In addition, the margins of error and noise on the *z*-axis are higher than those on the other axes [4]. The joint location information that was obtained from the depth data exhibited a similar tendency in terms of the margins of error [9]. Considering that 3D gestures involve motions in all directions, the study assigns weight values to the data exhibiting a low margin of error and less noise among multiple sequences, which are collected from multiple sensors in various directions. Previous studies confirmed that if a joint movement is closer to the *x*–*y* axis, it exhibits a lower margin of error and less noise [4,5]. Considering this observation, the viewpoint-weighted value *w^view^* of the element *n* of the input sequence *i* can be calculated using Equation (5):

(5) $$w_{i_{n}}^{view}=\max\left(\left|\frac{\left(i_{n}^{\prime }\times i_{n}\right)}{\left\|i_{n}^{\prime }\right\|\left\|i_{n}\right\|+\varepsilon }\right|,\alpha \right)$$

Herein, *i* is the sequence obtained by carrying out the primary differentiation on the location value sequence of a user and indicates the motion of a joint, and *i’* indicates the orthogonal vector connecting the position of the user’s joint and that of the camera; hence it is used to calculate the direction of the user's horizontal movement. The equation assigns weighted values by calculating the sine value of the two vectors. If the joint movement is parallel to the *x*–*y* plane, the value is 1, and if it shifts farther, the value approximates to 0. *ε* is a highly marginal constant value used to prevent calculation errors when the sizes of the two vectors are 0, and *α* is the minimum weight constant value that was set for the experiment.

If the following weight value is to be applied to the DTW,

(6) $$d_{weight}(i_{n},r_{m})=\sum_{j}^{J}\sum_{s}^{S}\frac{w_{i_{sn}^{j}}^{view}p_{sn}^{j}w_{r_{m}^{j}}^{motion}d(i_{sn}^{j},r_{m}^{j})}{\sum_{n=1}^{N}w_{i_{sn}^{j}}^{view}p_{sn}^{j}\sum_{m=1}^{M}w_{r_{m}^{j}}^{motion}}$$

the distance function *d* in Equation (4) is replaced with *d_weight_*, as illustrated in Equation (4). Herein, *i* is the input sequence, and *r* is the reference sequence of the database. *n* and *m* are the index numbers of each sequence (frame number), and the input sequence is the representative sequence of the multiple sequences obtained from the multiple sensors. *s* is the index number of a sensor, while *S* is the total number of sensors. Two types of weighted values apart for the viewpoint weight value are additionally used. *p* is the reliability of joint tracking; it can be calculated using Equation (7).

(7) $$p_{sn}^{j}=t_{sn}^{j}\frac{\sum_{n=1}^{N}t_{sn}^{j}}{N}$$

*t* is a value indicative of the tracking status, and *j* is the index number of joints. If the joint status is normally tracked, the value is 1; otherwise, the value is 0. *N* is the total frame number of the input sequence. Using Equation (7), the reliability of each joint was determined by considering the current tracking status of each joint and the overall tracking status. The Kinect software development kit (SDK) provides values indicative of the status when a joint is concealed by another object or when the tracking is unsuccessful. If a joint becomes concealed and is unsuccessfully tracked, the margin of error is excessively wide compared to the actual location of a joint. These wide margins of error can be reduced through the weighted values.

When a gesture requires the movement of numerous joints, no two joints are to be assigned an equal level of significance [10]. If higher weight values are assigned to a larger number of dynamic joints that involve a larger number of movements, their significance can be differentiated. The weight value *w^motion^*, which is used to differentiate the significance of joints, can be calculated using Equation (8):

(8) $$w_{r_{m}^{j}}^{motion}=\max\left(\frac{d(r_{m}^{j}-r_{m-1}^{j})}{\sum_{j}d(r_{m}^{j}-r_{m-1}^{j})},\beta \right)$$

The analysis of dynamic joints can be obtained if higher weight values are assigned to more dynamic joints in consideration of the movement quantity whenever a gesture is made. *β* is the minimum weight constant value that was set up for the experiment.

**Figure 5:**
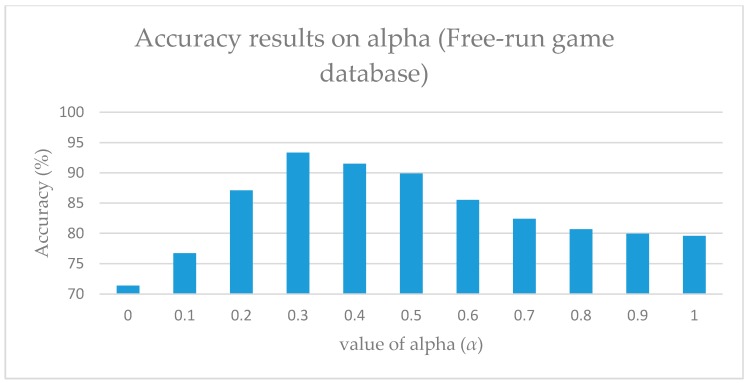
Accuracy results on alpha (*α*).

**Figure 6:**
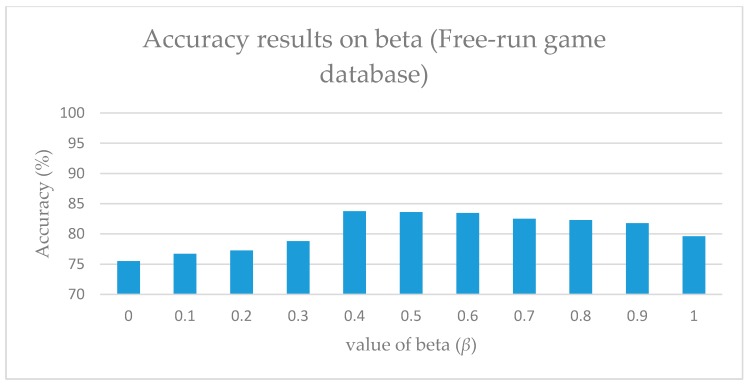
Accuracy results on beta (*β*).

As illustrated in Figure 5 and Figure 6, alpha indicates the contribution of the orthogonal value, and it aids in the setting of the maximum weight value. Therefore, by reducing the value of both alpha and beta, the effect of the weight can be adjusted. However, it was observed that if the minimum value of both alpha and beta are set to zero (0), the results obtained are completely defective. Moreover, this resulted in the consideration of the database’s characteristics during the adjustment.

Considering that it is not feasible to predict the optimal values of either alpha nor beta, each value was tested separately. For alpha, viewpoint-weighted DTW was used by setting the value of alpha from 0 to 1 for calculating the optimal value of alpha. Moreover, identical values were used for calculating the optimal values of beta. However, for beta, a motion-weighted DTW method was used. Figure 5 and Figure 6 illustrate the variations at particular values of these parameters. During the experiment, the Free-Run game database was used, and the optimal values were obtained at alpha = 0.3 and beta = 0.4. When using the G3D database, the optimal values were obtained at alpha = 0.2 and beta = 0.5.

## 4. Experiments

It is concluded that in addition to the general motion commands, the gestures used in games are appropriate for recognizing and utilizing dynamic and diverse movements. The proposed method was performed on two databases: the Free-Run game gesture database [18] and G3D [19]. To ascertain the performance of multiple sensor methods for 3D gestures, both the databases were reconstructed by capturing data using the three Kinect sensors. The main purpose of this research is to effectively combine data from multiple sensors to improve the recognition accuracy for three-dimensioanl gestures. To this end, we select the DTW algorithm for multiple sensors, since the DTW is better than HMM based approaches when time is a constraint [20] and easy to extend to multiple sensors.

### 4.1. Results of the Free-Run Game Gesture

The target gestures used in the experiment consist of 18 types, which are inspired by the ‘free-run’ game [18]. Among the targets, 6 types are meant to point toward directions, 8 types are combat motions, and 4 types are related to modes. Furthermore, the present experiment was carried out offline, wherein normalization of Kinect skeleton data was conducted by considering each sensor’s position.

**Figure 7:**
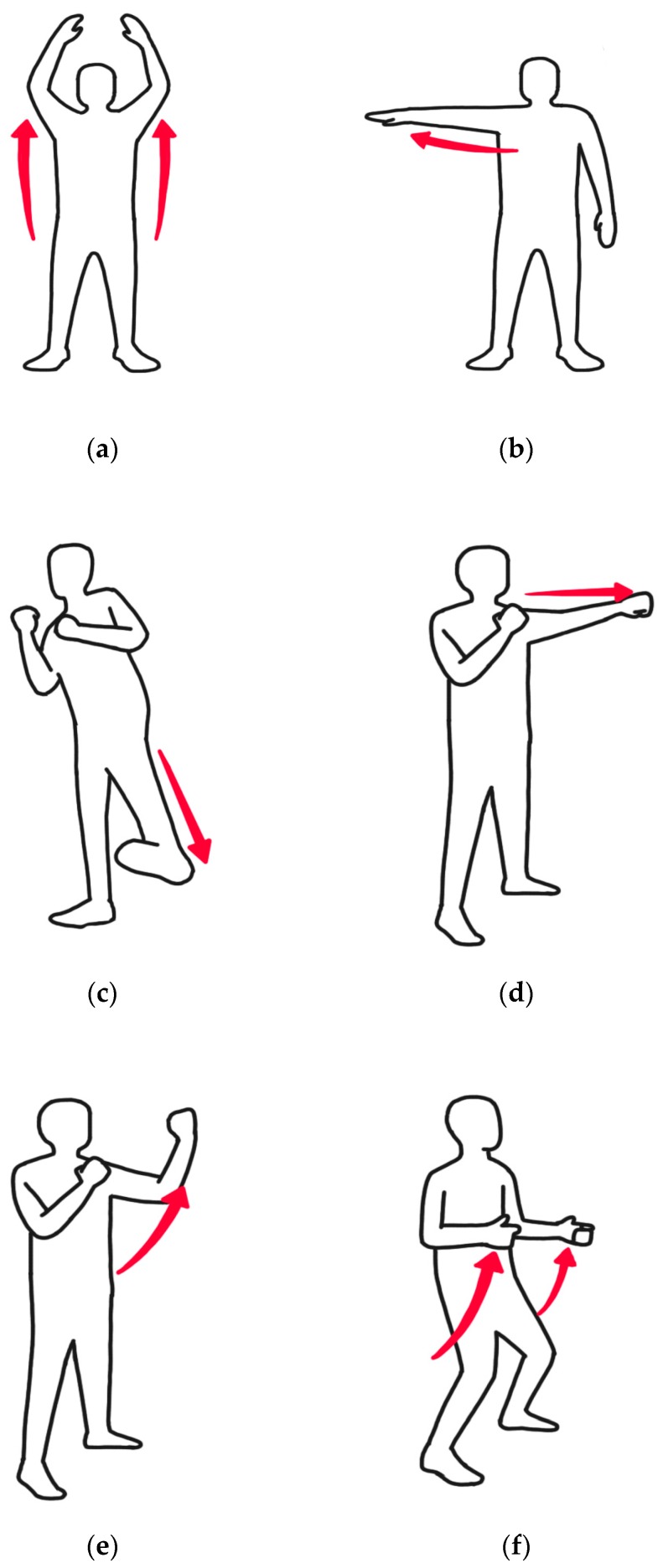
Examples of free-run game gestures: (**a**) hand flip; (**b**) point toward directions with one hand; (**c**) kick; (**d**) jab; (**e**) uppercut; (**f**) change weapons.

As illustrated in Figure 7, motion index number 1 is to flip both hands; numbers 2–7 are meant to point toward directions with one hand; numbers 8 and 9 are meant to kick; numbers 10–15 are meant to perform a jab, hook, or uppercut; and numbers 16–18 are meant to make big gestures with hands to change weapons or to reload. Index number 1 corresponds to Figure 7a, index numbers 2–7 correspond to Figure 7b, index numbers 8 and 9 correspond to Figure 7c, index numbers 10–15 correspond to Figure 7d,e, and index numbers 16–18 correspond to Figure 7f.

The database includes data on six individuals who performed each gesture 17 times. The gesture information is obtained from three Kinects installed at an interval of 120°, and each individual performed 18 gestures. Therefore, 1836 gestures were recorded in the database.

The beginning and ending parts of a gesture were manually excluded in advance. The (gesture recognition) performance was measured using the ‘1-nearest neighbor (1-NN)’ method. One sequence represents one motion, and multiple sequences, which were obtained from multiple sensors, were manually combined to minimize the margin of measurement errors and noise. Although there are marginal variations in identical gestures, these are due to handedness.

To verify the respective performance of the viewpoint-weighted method with multiple sensors and motion-weighted method with multiple sensors, the test was conducted using each of them separately and then by using a combination of both the methods. The 1-NN method requires a candidate for each class; therefore, the samples with the smallest inner-class distance were selected. The manually created representative sequence of each gesture was used to measure performance through the 1-NN method. The experimental results of the weighting methods are illustrated in Figure 4. The vertical g1 to g18 represent the input gesture types, while the horizontal g1 to g18 indicate the recognition results.

**Figure 8:**
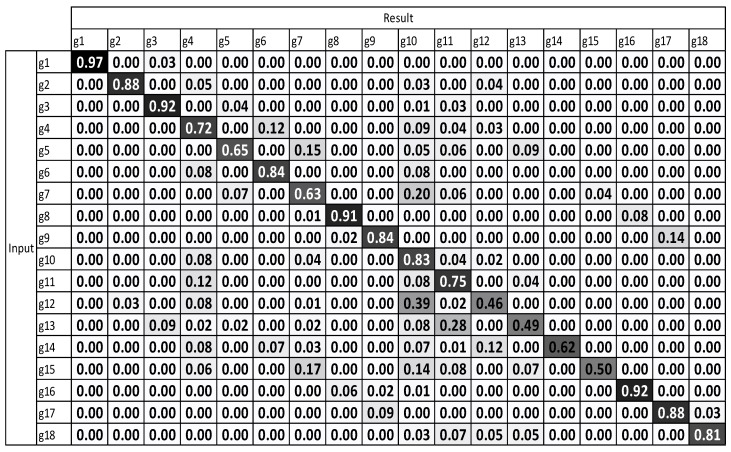
Result of standard DTW with a single sensor.

Figure 8 illustrates the low accuracy result in g12 to g15. These gestures are hook and uppercut, which are moved with “depth direction” and “draw an arch.” For deeper orthogonal movement, the gesture accuracy decreases with respect to others in standard DTW with one sensor. The average accuracy of a standard DTW with one sensor is 75.65%.

**Figure 9:**
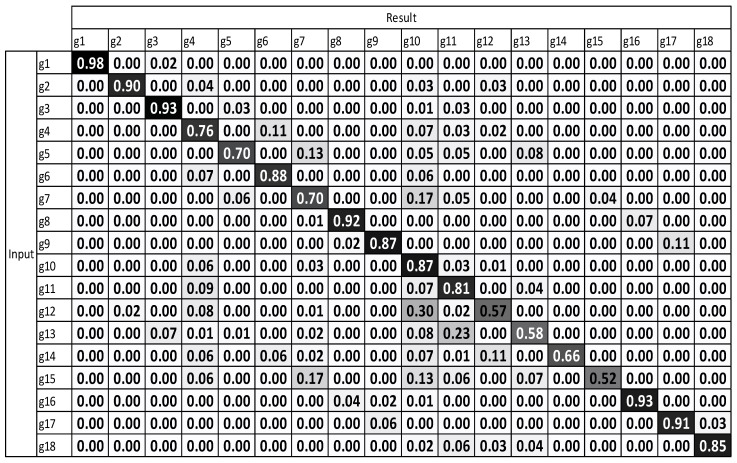
Result of standard DTW with multiple sensors.

**Figure 10:**
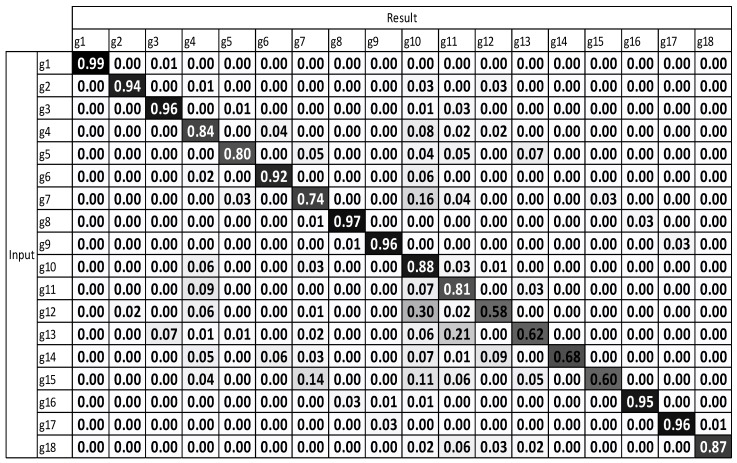
Result of motion-weighted DTW with multiple sensors.

Figure 9 and Figure 10 illustrate that after using multiple sensors, a large majority of the gestures were enhanced. The results demonstrate that the recognition performance was considerably enhanced.

The average accuracy of standard DTW with multiple sensors is 79.59%. The average accuracy of motion-weighted DTW with multiple sensors is 83.75%.

**Figure 11:**
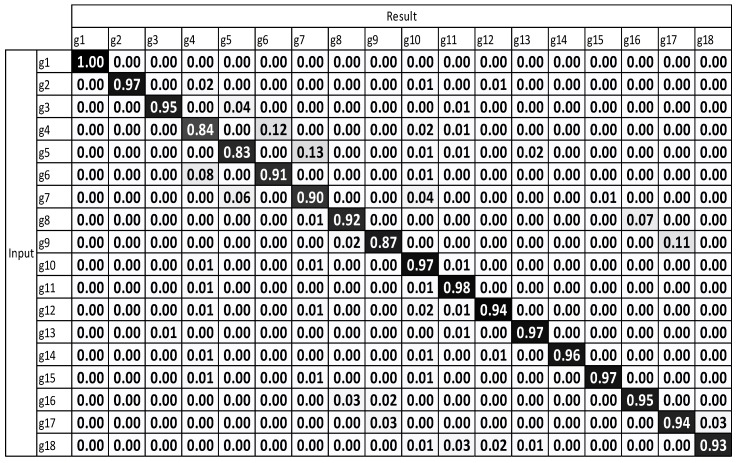
Result of viewpoint-weighted DTW with multiple sensors.

**Figure 12:**
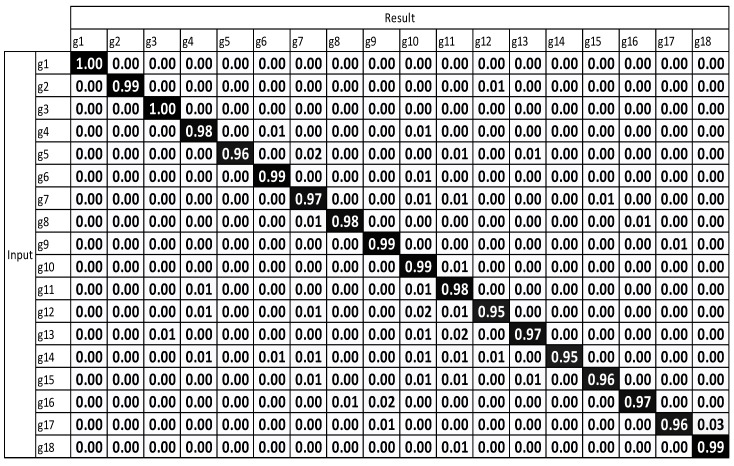
Result of fully-weighted DTW with multiple sensors.

The experimental results, which were determined using the standard DTW with one sensor (Figure 8), demonstrate that g12 to g15 exhibit the highest misrecognition rates (or the lowest recognition rates). g12 to g15 are the combating gestures of hook and uppercut. The standard DTW with multiple sensors and motion-weighted DTW with multiple sensors (Figure 9 and Figure 10) displayed an enhancement of the overall performance. However, g10–g15 underwent modest enhancements. The concerned gestures (g10–g15) are combat motions such as jab, hook, and uppercut, where the motions on the *z*-axis had a relatively larger number of characteristics. When the motion-weighted values were excluded and only the viewpoint-weighted values were taken into consideration, the accuracy of numerous gestures on the *z*-axis was enhanced significantly. The average recognition accuracy of the standard DTW with one sensor was measured at 75.65%, standard DTW with multiple sensors at 79.59%, motion-weighted DTW with multiple sensors at 83.75%, and viewpoint-weighted DTW with multiple sensors at 93.33%, as illustrated in Figure 11; the average recognition accuracy of the fully-weighted DTW with multiple sensors was measured at 97.77%, as illustrated in Figure 12.

### 4.2. Results on G3D Gesture

Numerous extant public action databases containing video sequences are available. However, for gaming that requires a variety of controls, commonly used action recognition databases are insufficient to cover the various movements of the user performed during the game. Public databases containing both video and skeleton data for sports and exercise motion actions exist; however, there are variations between performing an actual action and a gaming action for control [19].

**Figure 13:**
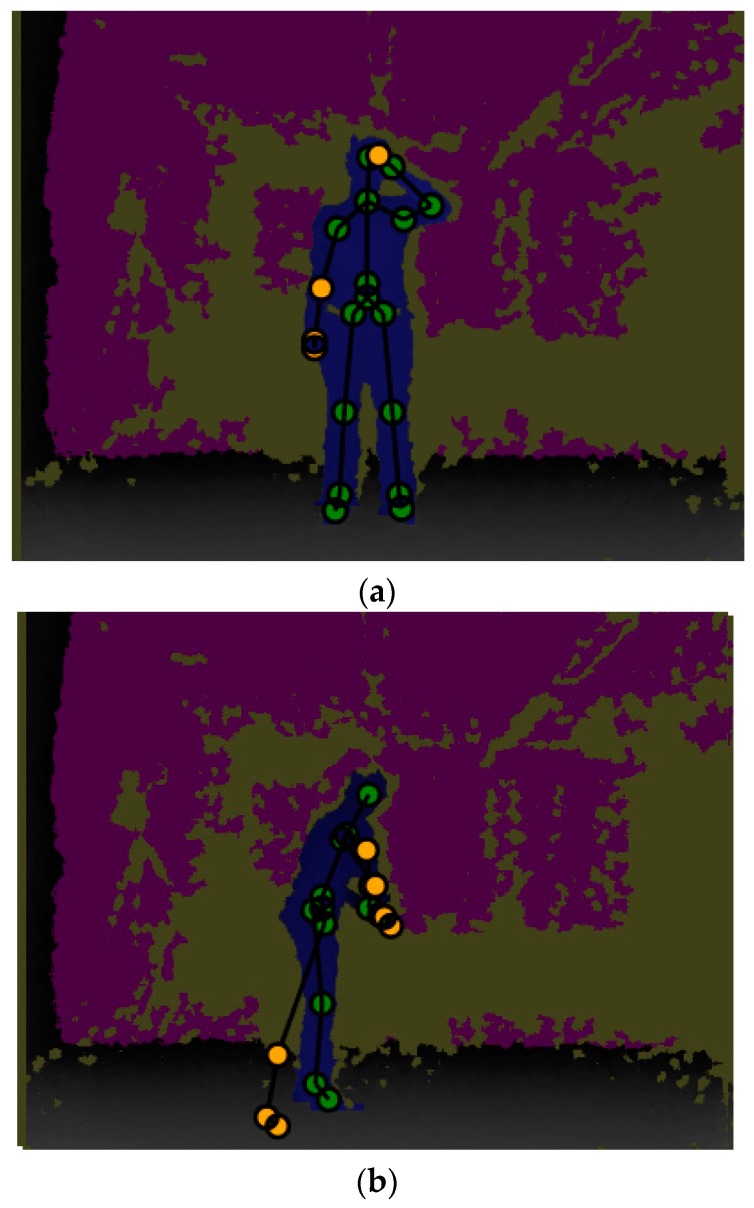
Example of the G3D database. (**a**) Shows forward captured data. It includes miss-tracked problem on left hand; (**b**) shows the golf gaming gesture. Due to sensor’s blind spot, there are mistracked joints on the left leg. The problem of mistracking on (**a**)’s both hands and (**b**)’s right hand are due to adjacent joints of both hands

The G3D database contains 20 gaming gestures (punch right, punch left, kick right, kick left, defend, golf swing, tennis swing forehand, tennis swing, backhand tennis serve, throw bowling ball, aim and fire gun, walk, run, jump, climb, crouch, steer a car, wave, flap, and clap). Moreover, these gestures were captured through a Kinect sensor in an indoor environment. In [19], the authors also encountered miss-tracked joints, which were due to the depth sensor’s limitation and various ranges of the gestures (as indicated in Figure 13). Therefore, the present method of using multiple sensors has provided an appropriate solution to this problem. Consequently, it was decided to select the G3D database. However, as a result of this selection, the present study encountered the challenge that it accepts datasets of only one sensor. Therefore, in applying this method, these datasets were recaptured using three Kinect sensors. Data capturing was carried out in the room where the Kinects were installed at intervals of 120°, and all the gestures were repeated 20 times by eight people.

Table 1 presents the overall results of each database. All the methods using multiple sensors demonstrate higher accuracy than that of a single sensor, albeit exhibiting negligible enhancement when no weights were used. As illustrated in the result figures presented in Section 4.1, the use of motion weights exhibited reasonable performance in motion where numerous joints could move, and the use of viewpoint weights exhibited high performance for gestures moving perpendicular to the sensors. As a result, the best results were obtained from both the databases when all the weights were used.

### 4.3. Time Cost and Comparison with Other Methods.

To obtain the value of the weight, it requires a constant range of time, and this has exhibited high accuracy compared to other methods that were used previously. Table 2 illustrates the calculated time cost. In the present method, the time costs depend on the number of targeted gestures and the length of the sequences carried out in a limited time.

## 5. Conclusions/Recommendations

In this study, which aims to develop a 3D motion recognition algorithm applicable to various environments that enables to expand the application of the CPS, the methods of the standard DTW with one sensor, motion-weighted DTW with multiple sensors, view-point-weighted DTW with multiple sensors, and fully-weighted DTW with multiple sensors are proposed and compared. The viewpoint-weighted DTW with multiple sensors enhances the performance by preventing joint measurement errors and noise due to the sensor’s measurement tolerance. The viewpoint-weighted DTW with multiple sensors effectively utilizes multiple sequences in the case of the gestures that are characterized mainly by motions on the *z*-axis. The motion-weighted DTW with multiple sensors enhances recognition performance by comparing multiple joint sequences effectively. Considering that the fully-weighted DTW uses multiple sensors and can recognize various gestures without directional or motional constraints, it is likely to be useful in various applications.

Considering that time cost depends on the size of the database and that it contains a substantially large number of gestures, there is a requirement for cost reduction. The present authors intend to overcome this challenge by using the matching algorithms in the gesture recognition systems in future research studies. It will perform better by pre-choosing the appropriate candidate for the gesture while analyzing the database by replacing the empirical value with the analyzed results. This will provide considerable cost reductions during gesture processing.

## Figures and Tables

**Table 1:** Accuracy results on each database.

Database	Standard DTW	DTW with Multiple Sensors	Motion-Weighted DTW	Viewpoint-Weighted DTW	Fully Weighted DTW
Free-run (18 gestures)	76.65%	79.55%	83.75%	93.33%	97.77%
G3D (20 gestures)	69.55%	73.50%	81.20%	89.95%	92.05%

**Table 2:** Average time cost for to make the results on each database.

Database	Standard DTW	DTW with Multiple Sensors	Motion-Weighted DTW	Viewpoint-Weighted DTW	Fully Weighted DTW
Free-run (18 gestures, average number of frames per each gesture is 180)	4.37 ms	11.61 ms	11.69 ms	29.86 ms	30.03 ms
G3D (20 gestures, average number of frames of each gesture is 90).	2.86 ms	7.86 ms	7.89 ms	19.41 ms	19.45 ms
